# Mice expressing an XRCC1 truncated protein are at increased risk for insulin resistance

**DOI:** 10.1080/20010001.2019.1603517

**Published:** 2019-04-25

**Authors:** Kavita Sharma, Jinzi Wu, Shu Xian Lee, Warren C. Ladiges, Jorming Goh

**Affiliations:** a Department of Comparative Medicine, School of Medicine, University of Washington, Seattle, WA, USA; b Gwinnett Medical Centre, 1000 Medical Centre Dr, Lawrenceville, GA, USA

**Keywords:** Insulin resistance, XRCC1 truncated protein mouse model, high fat diet, respiratory exchange ratio

## Abstract

Insulin resistance is a metabolic disorder that is highly prevalent in older populations. Mice expressing a truncated X-ray repair cross-complementing protein 1 (XRCC1tp) have normal repair of single-stranded breaks (SSBs) but are sensitive to alkylating agents. XRCC1tp mice thus provide a model to study perturbations in physiological function, such as metabolism, in the presence of normal DNA repair but attenuated XRCC1 activity. XRCC1tp male mice at six months of age fed a diet high in fat (lard) and sugar (sucrose) (HFSD) for three months showed a significant delay in glucose clearance, indicative of insulin resistance. These mice also had a decrease in respiratory exchange ratio, suggesting a change in the way fats and carbohydrates are used as a fuel source. Mechanisms for these observations are of interest, since there is a suggestion that XRCC1 is involved in glucoregulatory pathways, and XRCC1tp mice would provide an excellent model to pursue these studies in an age-related manner.

Disorders of metabolic function, such as insulin resistance, are highly prevalent in older populations. It has previously been shown that mice expressing a truncated X-ray repair cross-complementing 1 protein (XRCC1tp) are sensitive to alkylating agents but have normal DNA single-strand break (SSB) repair [Pettan-1, 2], suggesting that XRCC1 plays a role in mechanisms not related specifically to repair of SSB [3]. In an effort to investigate perturbations in metabolic function in the presence of normal SSB repair, but potential attenuated XRCC1 activity, cohorts of eight XRCC1tp and eight wildtype (wt) littermate male mice on a C57BL/6 background, were fed a diet high in fat (lard) and sugar (sucrose) (HFSD) for three months. The HFSD comprised 60% of kcalories from fat (F1850, Bio-Serv). All animals were kept in a specific pathogen-free facility with a 12-h light/12-h dark cycle. Monitoring food intake for three days once a month showed no difference in the amount of food consumed between groups.

The mean body weight at baseline was 30.4 gm for XRCC1tp mice and 31.7 gm for wt mice, which increased to 44.76 gm and 40.16 gm, respectfully, after 2 months with a significant cohort difference at p ≤ 0.05. However, this significance disappeared after 3 months, when mouse cohorts weighed 46 ± 4 g and 45 ± 5 g, respectively. The percent fat mass measured by quantitative magnetic resonance imaging (QMRI) increased in a manner that mirrored the increase in body weight.

An insulin sensitivity test was conducted at baseline and after three months on the HFSD diet. Following an overnight fast, mice were injected intraperitoneally with human insulin at 1.0 unit/kg body weight. Blood was obtained from the tail vein and glucose concentration measured with a glucometer at 0, 30, 60, and 90 min. At baseline, XRCC1tp mice had normal clearance of blood insulin similar to wt mice (Figure 1(a)). However, after 3 months on the HFSD, XRCC1tp mice had a significant delay in glucose clearance at 30 min after insulin injection (Figure 1(b)), indicating insulin resistance. This observation suggests that XRCC1 is normally associated with maintaining insulin sensitivity through some type of glucoregulatory mechanism.

**Figure 1.**
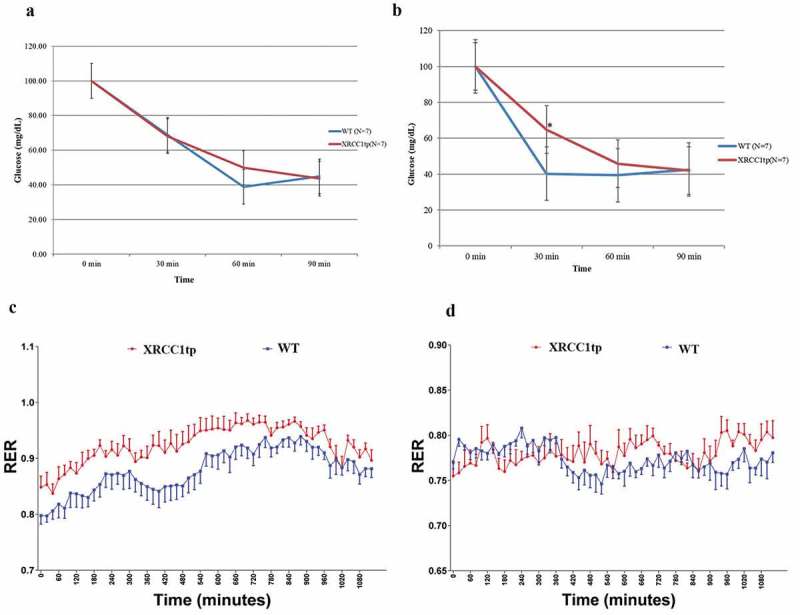
A high-fat sugar diet (HFSD) triggers insulin resistance and decreases the efficiency of energy utilization in XRCC1tp mice compared to wt littermates. (**a)** There were no differences in glucose levels after insulin injections at baseline between XRCC1tp and wt mice. **(b)** Significant differences did occur at the 30-min post-insulin injection time point, after 3 months on the HFSD, p ≤ 0.01. The bars represent the mean values for the number of animals in each group, and the error bars represent the SEM. **(c)** The respiratory exchange ratio (RER) at baseline, calculated from the ratio of VCO_2_/VO_2_ across 20 h, was significantly higher in XRCC1tp mice compared to wt littermates **(d)**, which disappeared after three months on the HFSD.

The respiratory exchange ratio (RER), calculated as a reflection of the volume of CO_2_ produced (VCO_2_ mL/kg/hour), relative to the volume of O_2_ consumed (VO_2_ mL/kg/hour), as measured at intervals of 20 min over 20 h using the Oxymax system (Columbus Instruments, OH), was significantly increased at baseline in XRCC1tp mice compared to wt littermates (Figure 1(c)). This difference disappeared after three months on the HFSD (Figure 1(d)). Both groups had a significant decrease in RER after three months on the high-fat diet, as analyzed by two-way ANOVA (p ≤ 0.01, F value of 1.7). Marvyn et al. [4], reported that RER values can be used to indicate the predominant fuel source used for energy, suggesting that an RER value of 0.70 aligns with fat, an RER of 0.85 aligns more with a mix of fat and carbohydrates, and a value of 1.00 or above aligns with carbohydrate. This would this suggest then that XRCC1tp mice were not able to utilize fat as an energy source as efficiently as wt mice before starting on the HFSD. However, after three months on the HFSD, XRCC1tp mice were forced to utilize fat more as an energy source but the extreme metabolic stress resulted in insulin resistance.

These observations suggest that XRCC1 may be involved in glucoregulatory pathways, and XRCC1tp mice would be a good model for further investigations.
